# A connectome-based neural correlate of pediatric ADHD hyperactivity–impulsivity symptoms

**DOI:** 10.3389/fpsyt.2026.1846942

**Published:** 2026-06-17

**Authors:** Jie Tao, Yue Wu, Ping Liu, Rong Wang, Ranran Gao, Dai Zhang, Qing Zhang, Feng Geng

**Affiliations:** 1 School of Mental Health and Psychological Science, Anhui Medical University, Hefei, China; 2 Department of Psychology and Sleep Medicine, The Second Affiliated Hospital of Anhui Medical University, Hefei, China; 3 Department of Radiology, The Second Affiliated Hospital of Anhui Medical University, Hefei, China; 4 Medical Imaging Research Center, Anhui Medical University, Hefei, Anhui, China

**Keywords:** attention deficit disorders with hyperactivity, child, connectome-based predictive modeling, functional connectivity, functional magnetic resonance imaging

## Abstract

**Background:**

This study examined the brain networks related to hyperactivity and impulsivity in children with Attention-Deficit/Hyperactivity Disorder (ADHD) and aimed to develop a resting-state functional connectivity marker that can predict symptom severity.

**Methods:**

A total of 44 children with ADHD (31 boys and 13 girls; mean age = 8.45 ± 1.52 years; range = 6–12 years) who met DSM-5 criteria were included. Resting-state fMRI data and clinical symptom scores were collected. Connectome-based predictive modeling (CPM) was used to predict hyperactivity–impulsivity symptoms based on whole-brain functional connectivity matrices. Symptoms were assessed using the SNAP-IV Parent and Teacher Rating Scales and the Conners Comprehensive Behavior Rating Scale. Model performance was tested with leave-one-out cross-validation and permutation testing.

**Results:**

The CPM model based on interregional functional connectivity successfully predicted hyperactivity–impulsivity symptoms (SNAP-IV parent and teacher ratings: r = 0.48, p = 0.001). The model also generalized well within the same dataset, as the selected functional connections significantly predicted hyperactivity–impulsivity scores on the Conners Parent Rating Scale (r = 0.49, p = 0.0009). Further analysis showed that stronger connectivity between the frontoparietal control network (FPN) and the dorsal attention network (DAN), and weaker connectivity between the FPN and the ventral attention network (VAN) and between the FPN and the somatomotor network (SMN), were related to symptom severity.

**Conclusions:**

Whole-brain functional connectivity is associated with hyperactivity–impulsivity symptoms in children with ADHD. The findings highlight the key role of FPN-related networks in these symptoms and provide a neural correlate that, pending further validation, represents a preliminary candidate for a neuroimaging marker.

## 1 Introduction

Attention-Deficit/Hyperactivity Disorder (ADHD) ranks among the most prevalent neurodevelopmental conditions, affecting approximately 8.0% of children and adolescents worldwide (1). The core symptoms in children with ADHD include inattention and hyperactivity-impulsivity. Its primary characteristic is a level of inattention and/or impulsivity-hyperactivity that is inconsistent with developmental level, accompanied by impairments in one or multiple areas such as learning or social functioning (2). Inattention symptoms are mainly manifested as difficulties in sustaining attention, increased distractibility, careless mistakes, and delayed task completion. Hyperactivity is characterized by excessive and purposeless motor activity, such as restlessness, frequent fidgeting, and difficulty remaining seated in situations that require quiet behavior. Impulsivity is typically reflected in difficulties inhibiting immediate responses, including interrupting others, difficulty waiting for turns, and acting without adequate forethought. Based on the predominance of symptoms, ADHD is commonly classified into three subtypes: predominantly inattentive type (ADHD-I), predominantly hyperactive–impulsive type (ADHD-HI), and combined type (ADHD-C). Among these, the combined type is the most frequently observed in clinical settings and involves the co-occurrence of inattention, hyperactivity, and impulsivity symptoms. ADHD is often accompanied by various comorbidities and adverse academic and social outcomes, with negative effects frequently persisting into adulthood and throughout the patient’s lifespan (3–7). Current research indicates that individuals with ADHD exhibit characteristics such as academic difficulties, strained family relationships, and peer interaction problems already in childhood (3). Particularly noteworthy is that individuals with ADHD have a higher propensity for substance abuse compared to the general population, a risk that is more pronounced in those comorbid with conduct disorder or antisocial personality disorder (8). Longitudinal studies show that individuals with ADHD face multiple challenges during adolescence and adulthood, including low academic and occupational achievement, reduced work capacity, metabolic disorders, emotional dysregulation, and even serious consequences such as suicidal tendencies (4, 9, 10). Epidemiological surveys confirm that ADHD can increase the risk of developing secondary mental disorders by 50%-300%, with substance use disorders, mood disorders, and anxiety disorders being particularly common (11–13). Furthermore, children with ADHD have a significantly elevated risk of developing antisocial and criminal behaviors. These behaviors often begin in childhood and are frequently mediated by conduct disorder (6).

Although ADHD causes widespread and persistent functional impairments at both clinical and societal levels, its current diagnosis primarily relies on reports of clinical symptoms from patients themselves, guardians, teachers, and other informants. While this symptom-based diagnostic model is a common approach in mental health disorders, it lacks objective diagnostic tools (2). The diagnostic methods commonly used in clinical practice, such as behavioral observations, subjective assessments, and symptom scales, hold some value but remain insufficient in terms of diagnostic specificity, accuracy, and early identification efficacy (14, 15). The core challenge in ADHD diagnosis stems from its high heterogeneity, manifested as significant individual differences in symptom combinations and severity, along with frequent comorbidity with other mental disorders. This not only increases diagnostic complexity but can also lead to misdiagnosis or delayed intervention (16). Particularly noteworthy is that ADHD symptoms in early childhood are often atypical and overlap with symptoms of other neurodevelopmental disorders, making differential diagnosis difficult (17). Research data indicate that there are no significant differences in functional impairment, treatment response, or prognosis between individuals who develop ADHD symptoms before age 7 and those who develop them at a later age. Furthermore, some high-functioning patients (e.g., those with high IQ, predominantly inattentive presentation, or those in highly structured environments) may not show clear functional impairment until middle to late elementary school years (15). The current multi-step diagnostic assessment process is time-consuming, and its key component, the clinical interview, is susceptible to the reliability of the informant (14). Although auxiliary methods like Continuous Performance Tests (CPT) and neuroimaging hold significant value for diagnostic verification, current clinical practice still lacks reliable, objective diagnostic indicators. Therefore, there is an urgent need to explore objective preliminary candidate based on neurobiology to improve the early identification and precise diagnosis of ADHD.

In recent years, advances in functional neuroscience techniques have provided important directions for the exploration of objective biomarkers for Attention-Deficit/Hyperactivity Disorder (ADHD). Electroencephalography (EEG), a classical neurophysiological technique, has made significant progress in detecting abnormal brain function and monitoring treatment effects in ADHD due to its high temporal resolution and non-invasive nature. Aydın et al. reported that brain network metrics derived from eyes-open resting-state EEG in boys with the combined subtype of ADHD (ADHD-C) could effectively distinguish brain functional states before and after methylphenidate treatment. Moreover, global functional connectivity across the whole brain was significantly increased after treatment, suggesting that EEG-based functional connectivity analysis may serve as an objective tool for monitoring treatment response and evaluating brain functional abnormalities in ADHD (18). In addition, an entropy-based quantitative EEG study conducted by Çetin et al. demonstrated that permutation entropy could accurately detect a significant reduction in cortical complexity in frontal and other cortical regions after methylphenidate treatment in children with ADHD. Furthermore, changes in EEG entropy values were closely associated with ADHD symptom severity and treatment improvement rates, further supporting the value of EEG-derived metrics in the quantitative assessment of ADHD symptoms (19). The above studies collectively demonstrate that EEG-based analyses of functional connectivity and cortical complexity can effectively capture core abnormalities in brain function associated with ADHD, providing important neurophysiological evidence for the objective assessment of the disorder. However, due to its limited spatial resolution, EEG is unable to precisely characterize functional connectivity patterns between specific brain regions and cannot adequately assess deep brain structures such as the striatum and thalamus.

In this context, functional connectivity has increasingly been recognized as an important biomarker for distinguishing different brain disorders. Blood oxygen level–dependent functional magnetic resonance imaging (BOLD-fMRI), with its superior spatial resolution, has therefore become a key technique for investigating abnormalities in brain functional connectivity in ADHD, offering new opportunities for advancing research in this field. Studies have shown that brain disorders, including Alzheimer’s disease, epilepsy, and ADHD, can impact the functional connectivity of neural networks (20). Accurately identifying the specific functional connectivity changes caused by a particular disease is considered a crucial task, as it can highlight the underlying mechanisms of the disorder. As a well-known non-invasive neuroimaging method, Blood Oxygen Level-Dependent functional Magnetic Resonance Imaging (BOLD-fMRI) effectively reveals brain abnormalities in individuals with ADHD by detecting the different magnetic properties of oxygenated and deoxygenated hemoglobin (21). Most current studies suggest that the occurrence of ADHD is related to abnormalities in the prefrontal cortex (22–24). Functional magnetic resonance imaging (fMRI) has revealed anatomical deficits and functional alterations in the prefrontal lobe, temporal lobe, and basal ganglia of individuals with ADHD (25). fMRI studies comparing adolescents and adults with ADHD to their unaffected peers have identified abnormal connectivity both within and between several key networks, including ventral attention regions, fronto-striatal-thalamic circuits, fronto-parietal networks, and the default mode network (26). Furthermore, evidence indicates that reduced connectivity in the right inferior frontal gyrus precedes the worsening of inattention symptoms, suggesting that functional hypoconnectivity may play a mechanistic role in the exacerbation and/or persistence of inattentive symptoms (27). Although existing studies have revealed significant functional connectivity abnormalities in the prefrontal cortex and related networks of ADHD patients, most literature focuses on the dimension of inattention. Previous studies have modeled attention deficits and demonstrated that functional connectivity between specific brain regions can be used to predict the development of inattentive symptoms (28, 29). By employing a fully cross-validated, data-driven methodology, investigators demonstrated that an individual’s level of sustained attention could be predicted from the overall strength of their functional brain networks. Specifically, they built a model describing the relationship between connection strength and task performance in a subset of participants who completed the gradual-onset continuous performance task (gradCPT) during functional MRI scanning, a task designed to assess sustained attention and inhibitory control (30–34). Analysis of the data revealed that a model constructed from these findings—designated the Sustained Attention Network (SAN)—was capable of forecasting behavioral performance in new individuals using their task-related functional connectivity. Moreover, the model could be generalized to resting-state functional connectivity, meaning that attention performance could be predicted using only resting-state connectivity features (29). In contrast, exploration of the neural correlate underlying the hyperactivity-impulsivity dimension remains limited. As a core symptom of ADHD, hyperactivity-impulsivity not only significantly impacts patients’ academic and social functioning but is also closely associated with substance use disorders and high-risk behaviors. Therefore, investigating its underlying neural basis is of great importance. Based on this, this paper uses functional connectivity as a starting point, focusing specifically on revealing the neural correlate of hyperactivity-impulsivity symptoms, aiming to provide new biological evidence for the precise diagnosis and intervention of ADHD.

However, many previous studies have primarily used correlation analyses to explore the relationship between alterations in local brain functional connectivity and behavioral or emotional problems in ADHD patients. Such methods have limitations in capturing complex brain network patterns. In recent years, supervised machine learning techniques have made significant progress in the field of individualized prediction. By establishing mapping relationships between high-dimensional features and clinical symptoms, their predictive performance is significantly superior to traditional univariate analysis methods.

This study employs Connectome-Based Predictive Modeling (CPM), a data-driven machine learning approach, to integrate multimodal neuroimaging features and clinical symptom data to build a model capable of predicting the core hyperactivity-impulsivity symptoms in children with ADHD. CPM represents a data-driven approach capable of identifying connectivity patterns across an individual’s whole-brain connectome, which can then be used to predict disease status, symptom severity, or cognitive performance (28, 29, 35, 36). We collected neuroimaging and behavioral data from 54 children with ADHD recruited through a psychiatry outpatient clinic, specifically to test whether hyperactivity-impulsivity symptoms could be predicted. Based on the SNAP-IV Parent and Teacher Rating Scales and the Conners Comprehensive Behavior Rating Scales, we hypothesized that specific neural network features could effectively predict an individual’s hyperactivity-impulsivity severity. To verify the model’s robustness, this study further used internal cross-validation to assess its predictive accuracy. Through this methodological framework, we aim to uncover the neural basis of hyperactivity-impulsivity symptoms and provide new objective evidence for the precise diagnosis of ADHD.

## 2 Materials and methods

### 2.1 Participants

From October 1, 2024, to June 30, 2025, a total of 54 children with attention-deficit/hyperactivity disorder (ADHD) who met the inclusion criteria were recruited from the outpatient clinic of the Department of Psychology at the Second Affiliated Hospital of Anhui Medical University (age 6–12 years). All participants were right-handed and had no history of neurological or psychiatric conditions. Written informed consent was obtained from their guardians prior to the study. Inclusion Criteria: 1. Aged 6–12 years. 2. Provided signed informed consent from both the child and guardian. 3. Ability to cooperate and complete the required assessments. 4. Met the diagnostic criteria for ADHD as defined by the Diagnostic and Statistical Manual of Mental Disorders, Fifth Edition (DSM-5). Exclusion Criteria: 1. Current or regular use (within the last 3 months) of any medication that could potentially influence central nervous system function or brain connectivity (e.g., antidepressants, antipsychotics, anxiolytics, sedative-hypnotics, corticosteroids, etc.), or participation in any other clinical trial/treatment. 2. Inability to cooperate with the examinations or complete the experiment. 3. Presence of severe systemic diseases, including cardiac, hepatic, pulmonary, or renal conditions. 4. History of epilepsy, traumatic brain injury, or brain surgery. 5. History of other psychiatric disorders or organic brain diseases affecting cognitive function. 6. Contraindications for MRI scanning (e.g., implanted ferromagnetic metal devices, cardiac pacemaker). 7. A score below 80 on the Wechsler Intelligence Scale. The ADHD sample included children across three subtypes: ADHD-I, ADHD-HI, and ADHD-C. During data analysis, 9 of the 54 initially enrolled patients were excluded due to incomplete resting-state fMRI data or excessive head motion. Resting-state fMRI analysis was therefore performed on 45 patients (32 boys; mean age = 8.34 ± 1.54 years). One patient’s clinical assessment data was excluded due to incomplete data collection. Consequently, psychological assessment data analysis included 44 patients.

### 2.2 Neuropsychological assessment

We used the SNAP-IV Parent and Teacher Rating Scales and the Conners Comprehensive Behavior Rating Scales (Parent Version) to assess hyperactivity-impulsivity symptoms in patients. The SNAP-IV scale is based on the ADHD diagnostic criteria outlined in the Diagnostic and Statistical Manual of Mental Disorders (DSM), and is commonly used to assess children’s behavioral symptoms across the core domains of inattention, hyperactivity, and impulsivity. The Conners Parent Rating Scale is an assessment tool completed by parents or primary caregivers based on the child’s daily behavior. It aims to systematically and quantitatively evaluate various behavioral problems in children and adolescents aged 6 to 18, including: core symptoms of ADHD, conduct problems, oppositional defiant behaviors, emotional issues (such as anxiety, fear), psychosomatic concerns (such as headaches, stomachaches), and a hyperactivity index, among other areas. Unlike the SNAP-IV, which focuses specifically on DSM diagnostic criteria, the Conners scale covers broader dimensions and provides a more comprehensive behavioral profile. In addition, participants’ socioeconomic status was assessed using the MacArthur Scale of Subjective Social Status (community version). In this measure, participants were presented with a 10-rung ladder representing the social hierarchy in Chinese society. The top of the ladder (10th rung) represents individuals with the highest income, highest level of education, and most respected occupations, whereas the bottom of the ladder (1st rung) represents individuals with the lowest income, lowest educational attainment, and least respected or no occupations. Participants were asked to place an “X” on the rung that best represented their family’s position in Chinese society. The selected rung score was used as an indicator of subjective socioeconomic status.

### 2.3 Functional magnetic resonance imaging data acquisition

Resting-state MRI data were collected from all participants at the hospital using a 3.0T scanner (MAGNETOM Vida, Siemens Healthineers, Munich, Germany). High-resolution T1-weighted structural images were obtained with a voxel size of 1×1×1 mm and a slice thickness of 1.00 mm for precise anatomical registration during image processing. Resting-state fMRI data for functional connectivity analysis were acquired using the following parameters: TR = 2400 ms, TE = 25 ms, matrix size = 64×64, FOV = 192 × 192 mm², slices = 48, voxel size = 3×3×3 mm, and 250 volumes. During scanning, foam padding was used to minimize head motion, and participants were instructed to lie still, keep their eyes open, and refrain from engaging in any specific mental activity. The study was conducted in accordance with the ethical principles of the Declaration of Helsinki, and written informed consent was obtained from the guardians of all participants prior to the experiment.

### 2.4 Functional magnetic resonance imaging data preprocessing

All fMRI data were preprocessed using the WhiteMatter toolkit (https://github.com/jigongjun/Neuroimaging-and-Neuromodulation), which integrates functions from AFNI (37), SPM12, and FSL. The preprocessing pipeline consisted of several steps: removal of the first 5 volumes, despiking, slice timing correction, and realignment. Functional images were then co-registered to structural images, which were subsequently segmented into gray matter, white matter, and CSF. We then regressed out 27 nuisance signals, including mean signals from white matter, CSF, and global brain, along with 24 head motion parameters (the 6 motion parameters, their derivatives, and quadratic terms). We are aware of the debate that global signal regression may introduce spurious negative correlations, but we followed the original CPM pipeline and kept this step for consistency. Finally, spatial smoothing was applied using a 4 mm FWHM Gaussian kernel, followed by temporal band-pass filtering between 0.01 and 0.1 Hz; and spatial normalization to the Montreal Neurological Institute (MNI) space using the matrices generated from structural image segmentation and the DARTEL algorithm in SPM12. If head motion during scanning exceeded 3 mm in translation or 3° in rotation, all images from that session were discarded. Among the 54 participants, nine were excluded due to incomplete resting-state fMRI data or excessive head motion.

### 2.5 Whole-brain parcellation and functional connectivity estimation

The Yeo functional network parcellation scheme was applied to divide the preprocessed resting-state fMRI data of each participant into 80 functionally defined brain regions (38, 39). Based on the MNI coordinates provided by the Yeo atlas, each region was localized in the fMRI data and the blood oxygen level–dependent (BOLD) signal time series was extracted. The region in the atlas was defined as a sphere centered at the MNI coordinate, with a 6 mm radius (40). Specifically, the mean BOLD signal for each region was obtained by averaging the time series of all voxels within that region. Pearson’s correlation coefficients (r) were then calculated between the time series of every pair of brain regions to quantify functional connectivity strength. This procedure generated an 80 × 80 symmetric functional connectivity matrix for each participant, in which each element represents the connectivity strength (edge) between two brain regions (nodes).

### 2.6 Connectome-based predictive modeling

**Figure 1:**
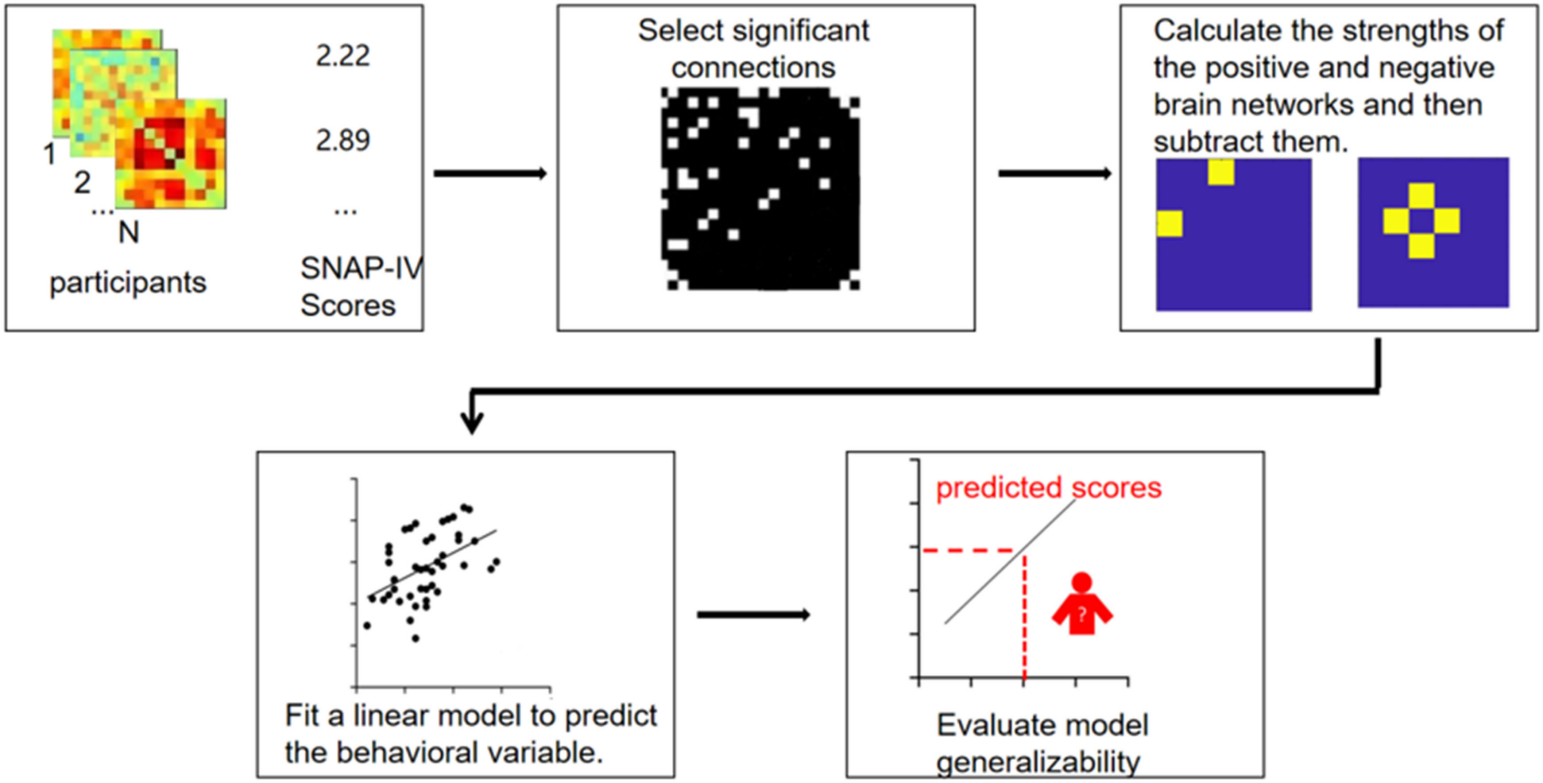
Schematic overview of the CPM construction process. This image illustrates how the connectome-based predictive modeling (CPM) framework is built. CPM, connectome-based predictive modeling.

**Figure 1** presents the schematic representation of the steps of CPM. First, to build the model, we employed a leave-one-out cross-validation (LOOCV) procedure. The data were divided into a training set (n-1 participants) and a test set (the remaining participant). This process was repeated until every participant had served as the test case once. During feature selection, for all participants in the training set, each edge of the functional connectivity matrix was correlated with the hyperactivity-impulsivity scores. Following established practices in the field and referencing previous studies that employed similar functional connectivity approaches (41), we applied a statistical threshold of p < 0.001 to identify significant connections. The correlation matrix was then converted into functional connectivity indicators, including inter-regional functional connectivity strength, by retaining only those connections that survived the threshold. Separate positive and negative networks were constructed based on this p-threshold. The positive network comprised edges with significant positive correlations, and the negative network comprised edges with significant negative correlations. The strength of each network was calculated for each individual. The whole-brain functional connectivity model was a combined model. Its network strength was calculated by subtracting the negative network strength from the positive network strength. A linear model was then fitted within the training set to relate this combined network strength to the hyperactivity-impulsivity scores. In the prediction phase, the regression coefficients obtained from the training model were applied to the held-out test participant to predict their hyperactivity-impulsivity score. This entire procedure was iteratively repeated until every participant had been the test case. Consequently, this CPM technique generated a predicted hyperactivity-impulsivity score for each participant. The Pearson correlation (rp) between these predicted scores and the observed scores was then computed to serve as a measure of model performance. A permutation test was conducted by randomly shuffling the observed ADHD hyperactivity-impulsivity scores. Following previous CPM studies, statistical significance was evaluated using 1000 permutation iterations to estimate the null distribution. This shuffled CPM-LOOCV procedure was repeated 1000 times to construct a null distribution, which was used to evaluate whether the model’s predictive performance was significantly better than chance. Additionally, to investigate the potential influence of demographic and motion-related confounding factors, we conducted supplementary sensitivity analyses using partial correlations instead of Pearson correlations in the feature selection step, controlling for age, sex, and head motion (mean framewise displacement).

### 2.7 Internal cross-validation

To test whether the functional connectivity features identified based on the SNAP-IV scale could generalize to predict scores from other clinical symptom assessments, we applied them to predict the hyperactivity/impulsivity factor scores of the Conners Parent Rating Scale. Model performance was evaluated using leave-one-out cross-validation (LOOCV). Specifically, in each iteration, data from n-1 subjects formed the training set, and the remaining single subject served as the test sample. First, using the training set data, a multiple linear regression model was built to establish the relationship between the network strength (derived from the SNAP-IV-based features) and the Conners hyperactivity/impulsivity scores, yielding regression coefficients (β). Subsequently, this resulting regression equation was applied to the left-out subject to predict their hyperactivity/impulsivity score. This entire procedure was repeated until every subject had been left out as the test sample once. Finally, we used Pearson correlation analysis to assess how well the predicted scores matched the actual observed scores, providing a measure of the model’s internal predictive performance. The statistical significance of this prediction performance was verified against random chance through a permutation test with 1000 iterations. The same covariate adjustment was applied during the prediction stage in the supplementary analysis.

### 2.8 External cross-validation

External validation sample. An independent external validation cohort was recruited from the same hospital using identical recruitment procedures, inclusion/exclusion criteria, and scanning parameters as the primary sample. To ensure temporal independence and avoid any overlap, recruitment for this cohort took place from August 2025 to January 2026, whereas the primary cohort had been enrolled between October 2024 and June 2025. The external validation sample comprised 18 children (15 boys, 3 girls) with a mean age of 9.06 years (SD = 1.55) and a mean score of 99.56 on the Intelligence Scale (SD = 8.19).

To assess the generalizability of the functional connectivity features identified using the SNAP-IV scale, we applied the whole-brain functional connectivity model trained on the training set to this external validation dataset. Statistical significance of the predictions was determined through 1,000 permutation tests to evaluate whether the results were significantly above chance level.

## 3 Results

A total of 44 children with ADHD were included in the analysis (mean age 8.45± 1.52 years; 31 boys, 13 girls). Among them, 42 subjects completed both the SNAP-IV and Conners Parent Rating Scales. Specifically, all 44 participants had valid SNAP−IV data, and 42 had valid Conners data (the remaining 2 participants missed the Conners scale). In our sample, 35.56% of mothers had a high school education or below, 26.66% had a junior college degree, 35.56% had a bachelor’s degree, and 2.22% had a master’s degree or above. For fathers, 13.33% had a high school education or below, 28.89% had a junior college degree, 55.56% had a bachelor’s degree, and 2.22% had a master’s degree or above. Descriptive statistics for demographic and clinical variables are summarized in **Table 1**.

**Table 1:** Demographic and clinical characteristics.

Characteristic	Mean (SD) or N (%)
Sex	
Girls	13(29.5)
Boys	31(70.5)
Age(years)	8.45 ± 1.52
Socioeconomic Status (SES)	4.96 ± 1.30
Intelligence Scale	99.37 ± 13.98
Hyperactivity-Impulsivity Score	15.14 ± 4.78

### 3.1 Localization of key brain regions and functional networks

**Figure 2:**
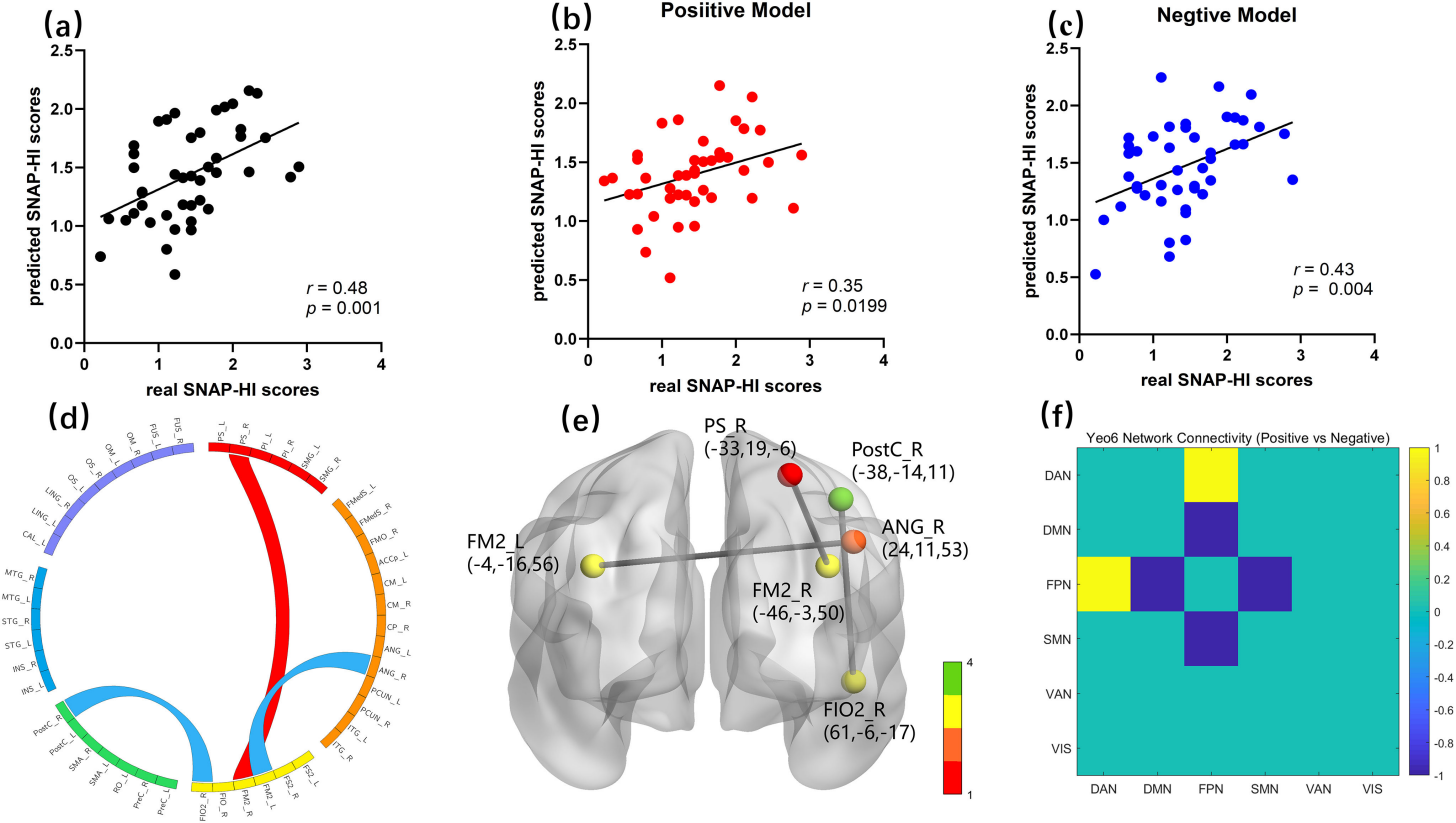
Prediction models and contributing connections. **(a)** Scatter plots show the correlation between the observed SNAP-IV hyperactivity/impulsivity scores and the predicted scores generated by the combined model.Correlations between real SNAP-IV hyperactivity/impulsivity subscale scores and predicted SNAP-IV hyperactivity/impulsivity subscale scores in **(b)** positive model and **(c)** negative model. **(d)** shows the positive and negative connections predicting hyperactivity/impulsivity scores. **(e)** Views of the network edges and nodes on a glass brain. **(f)** The contribution of each brain network to the prediction of hyperactivity-impulsivity symptoms.

Model analysis showed that the functional connections between the right middle frontal gyrus and the right superior parietal gyrus, between the left middle frontal gyrus and the right angular gyrus, and between the right orbitofrontal cortex and the right postcentral gyrus played important roles in predicting hyperactivity and impulsivity symptoms in ADHD (**Figure 2e**). These connections mainly involved interactions among the frontal, parietal, orbitofrontal, and somatosensory regions. At the network level, the positive connections were mainly distributed between the frontoparietal control network (FPN) and the dorsal attention network (DAN), while the negative connections were mainly distributed between the FPN and the ventral attention network (VAN) and the somatomotor network (SMN; see **Figure 2d**). These findings suggest that the imbalance between executive control and attention regulation systems may be an important neural basis of hyperactivity and impulsivity symptoms in ADHD, which is consistent with previous studies reporting abnormal functional organization in the FPN.

### 3.2 Model predictive performance for the SNAP-IV scale

**Figure 3:**
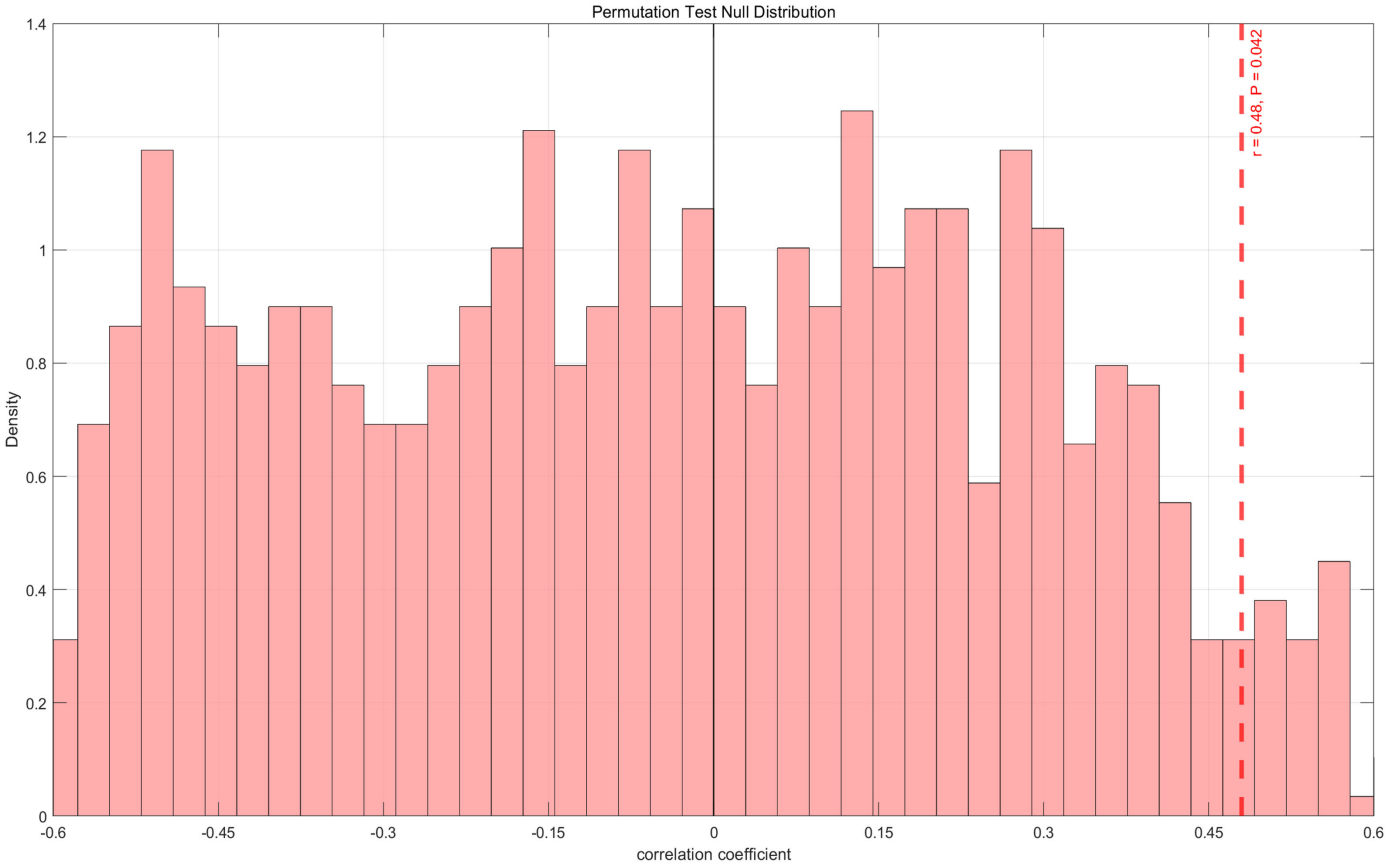
The permutation distribution of correlations for the combined brain network model.

We applied a connectome-based predictive modeling (CPM) framework using resting-state functional connectivity to develop a whole-brain model for ADHD. The model significantly predicted the hyperactivity/impulsivity subscale scores of the SNAP-IV (r = 0.48, 95% CI [0.21, 0.68], p = 0.001; 1000 permutation tests, p = 0.042, **Figures 2a–f**). To visualize the permutation-based significance test, the permutation distribution of the correlations for the combined brain network model is shown in **Figure 3**. When age, sex, and head motion were included as covariates in a supplementary analysis, the predictive correlation remained similar (r = 0.4998, p<0.001), indicating that the main findings were not driven by these potential confounds. Further analysis showed that both the positive and negative network models achieved significant prediction accuracy (positive model: r = 0.35, p = 0.0199, **Figure 2b**; negative model: r = 0.43, p = 0.004, **Figure 2c**). Among them, the negative model showed a better predictive performance. These results indicate that individual differences in hyperactivity and impulsivity may be closely related to reduced connectivity in specific brain networks.

### 3.3 Validation of generalization to the Conners scale

**Figure 4:**
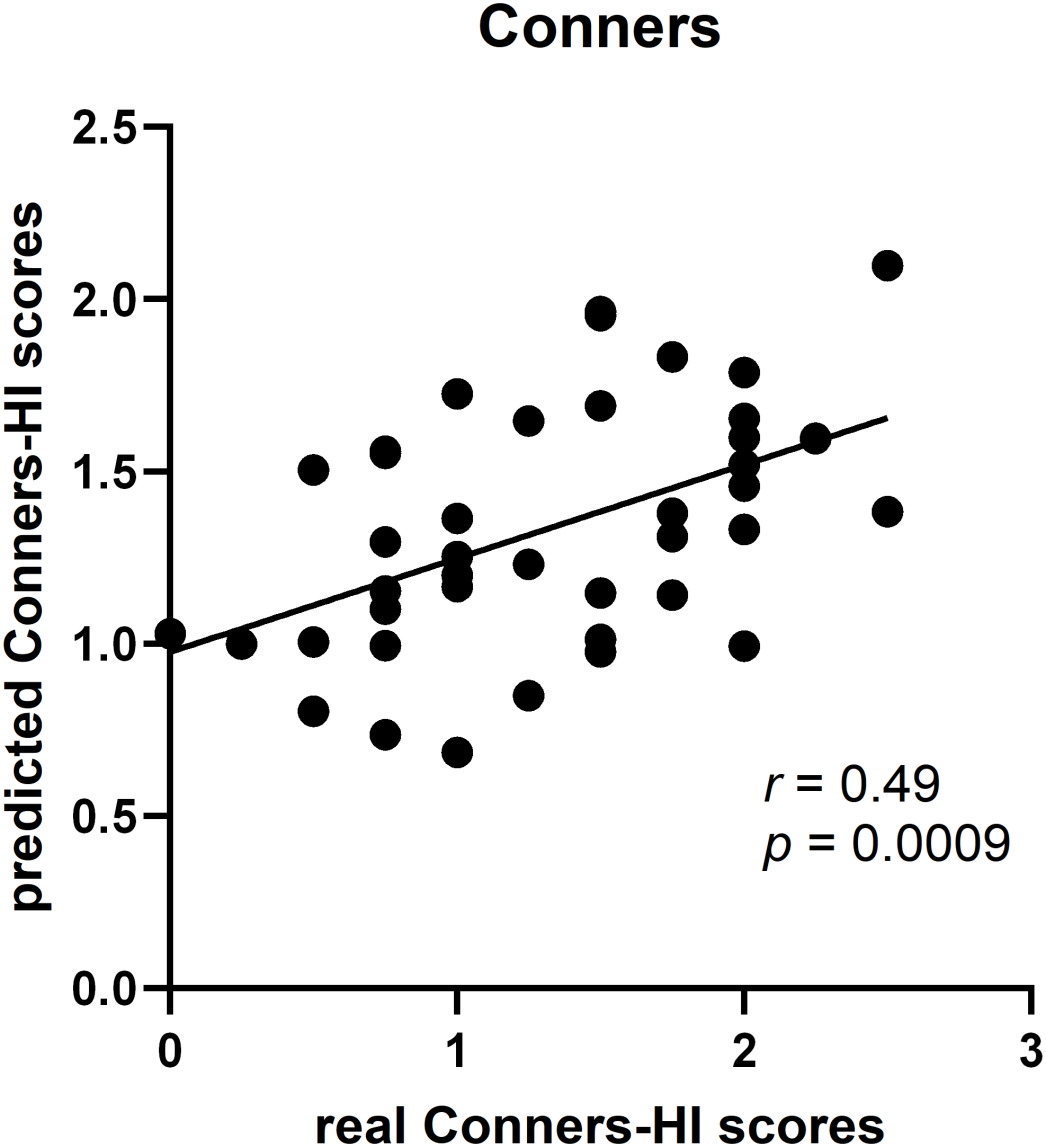
The correlation between the combined model’s predicted Conners hyperactivity/impulsivity scores and the observed scores on the Conners scale.

After confirming that resting-state functional connectivity could predict SNAP-IV hyperactivity/impulsivity scores, we further examined whether the selected features from this model could predict other behavioral scales. The results showed that the whole-brain functional connectivity model trained on SNAP-IV data also significantly predicted the Hyperactivity Index scores from the Conners Parent Rating Scale (r = 0.49, p = 0.0009; 1000 permutation tests, p = 0.002, **Figure 4**). After also including covariates in the supplementary analysis, the result was r = 0.55(p<0.001). This finding suggests that the model based on resting-state functional connectivity not only identifies neural patterns associated with ADHD hyperactivity and impulsivity symptoms but also demonstrates good generalization ability across different assessment scales. **Figure 4** shows the correlation between predicted and observed scores.

### 3.4 External cross-validation of the prediction model

**Figure 5:**
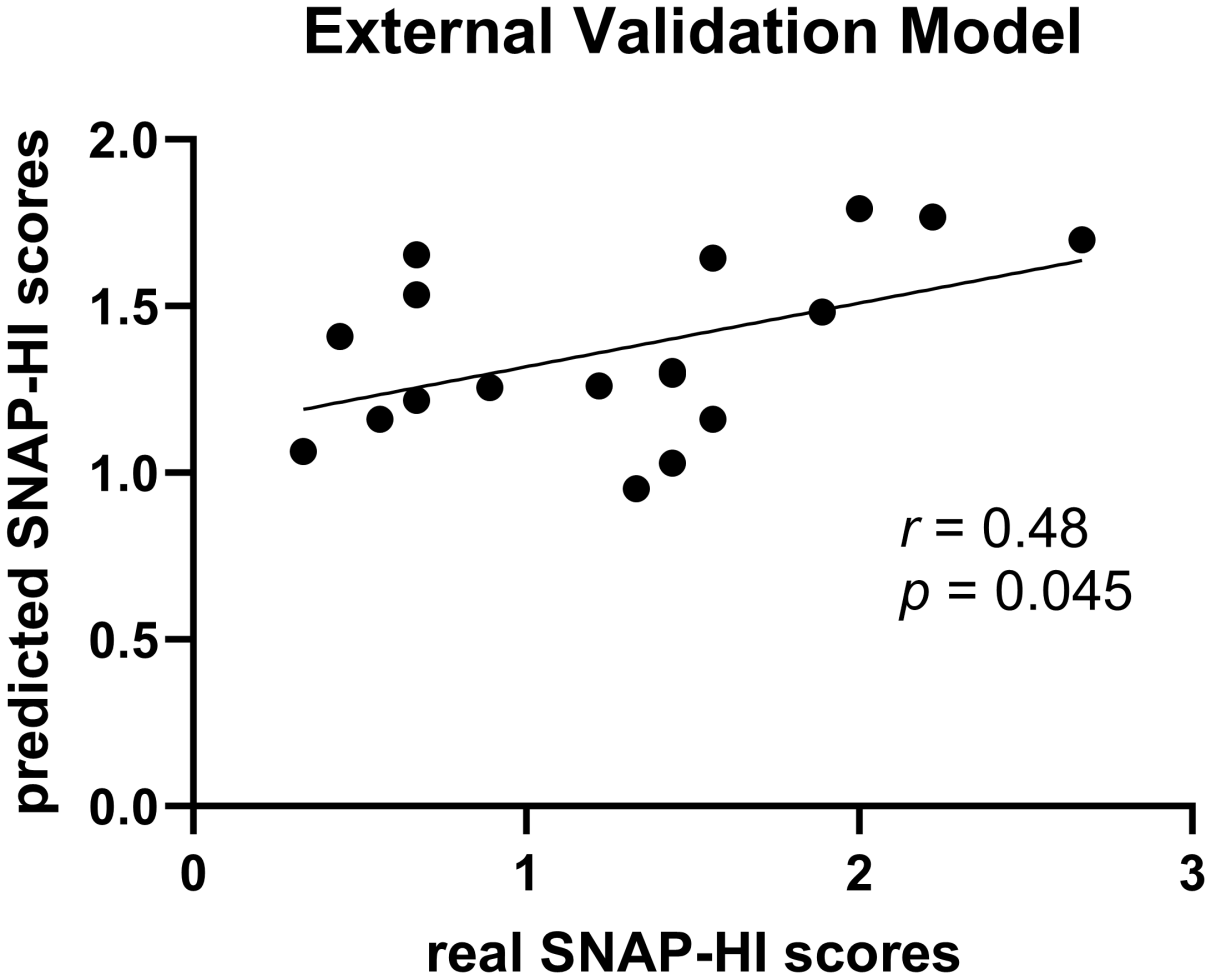
The correlation between the model’s predicted SNAP-IV hyperactivity/impulsivity scores and the observed scores in the external dataset.

After validating the model’s predictive performance on the Conners scale, we further evaluated its generalizability on an independent external dataset (n = 18). The whole-brain functional connectivity model trained on the SNAP-IV scale was applied to this separate sample. Results showed that the model significantly predicted hyperactivity-impulsivity scores in this external dataset (r = 0.48, p = 0.045; 1000 permutation tests, p = 0.007). These findings indicate that the resting-state functional connectivity–based model exhibits cross-dataset generalizability in the external dataset. **Figure 5** illustrates the correlation between predicted and observed scores in the external dataset.

## 4 Discussion

In this study, we successfully constructed a whole-brain functional connectivity model to predict hyperactivity/impulsivity symptoms in children with attention-deficit/hyperactivity disorder (ADHD) using the connectome-based predictive modeling (CPM) approach. The results showed that the key predictive edges were mainly located between the right middle frontal gyrus and the right superior parietal gyrus, the left middle frontal gyrus and the right angular gyrus, and the right orbitofrontal cortex and the right postcentral gyrus. At the network level, the positive network was primarily distributed between the frontoparietal control network (FPN) and the dorsal attention network (DAN), whereas the negative network was mainly distributed between the FPN and the ventral attention network (VAN) as well as between the FPN and the somatomotor network (SMN). Further analysis revealed that the model could significantly predict hyperactivity/impulsivity scores on the SNAP-IV (r = 0.48, p = 0.001) and effectively predict the hyperactivity index on the Conners’ Parent Rating Scale (r = 0.49, p = 0.0009). Additional sensitivity analyses demonstrated that the predictive performance was robust across different feature selection thresholds and remained stable after controlling for potential confounds such as age, sex, and head motion. These findings suggest that resting-state whole-brain functional connectivity patterns contain stable neural representations associated with hyperactivity/impulsivity symptoms in ADHD.

This study found that functional connectivity among frontal and parietal regions plays a key role in predicting hyperactivity/impulsivity symptoms, which is consistent with previous literature. Prior research has indicated that the middle frontal gyrus and orbitofrontal cortex are important for executive function and impulse control, and their functional abnormalities may lead to difficulties in behavioral inhibition and increased reward sensitivity (24). The superior parietal gyrus and angular gyrus, as key nodes of the attention network, are considered closely related to sustained attention and cognitive flexibility (42). At the network level, this study found that increased FPN–DAN connectivity was associated with hyperactivity/impulsivity symptoms, whereas decreased FPN–VAN and FPN–SMN connectivity may reflect insufficient top-down control and heightened sensitivity to external stimuli. These findings are highly consistent with previous studies on functional connectivity abnormalities in ADHD and further emphasize the unique neural correlate underlying the hyperactivity-impulsivity dimension (43, 44).

Our model revealed that the severity of hyperactivity-impulsivity symptoms is closely associated with increased connectivity between the FPN and DAN, as well as decreased connectivity between the FPN and VAN and between the FPN and SMN. This finding provides important clues for understanding the neural correlate of hyperactivity-impulsivity symptoms in ADHD. The FPN, as the brain’s “control hub,” is primarily responsible for higher-order executive functions and behavioral regulation, whereas the DAN is involved in top-down, goal-directed allocation of attention (45). We observed that the enhanced FPN–DAN connectivity may reflect a neural compensatory mechanism: in children with more severe symptoms, the brain may recruit additional cognitive control resources to suppress inappropriate behavioral impulses and maintain task focus. This compensatory “overexertion” is manifested in functional connectivity as increased coupling strength between key networks (46). While our findings show enhanced FPN–DAN connectivity, the interpretation as a compensatory mechanism is tentative. Future studies using longitudinal designs or task-based fMRI paradigms are needed to directly test whether this increased connectivity indeed reflects recruitment of additional cognitive control resources in children with more severe symptoms.

On the other hand, the reduced FPN–VAN connectivity has important theoretical implications. The VAN is responsible for bottom-up, stimulus-driven attention capture and for rapid responses to novel stimuli (47). Under normal conditions, the FPN exerts regulatory control over the VAN to suppress irrelevant stimuli that may interfere with ongoing tasks. We found that FPN–VAN functional connectivity was weakened, which is highly consistent with the inhibitory control deficit theory of ADHD, suggesting that impaired FPN regulation of the VAN makes patients more susceptible to external, unexpected stimuli, resulting in distractibility and impulsive behavior (42). At the same time, decreased FPN–SMN connectivity may reflect insufficient regulation of the sensorimotor system by the cognitive control network, leading to overactive motor behaviors, such as restlessness and frequent small movements (48, 49). These findings not only deepen our understanding of the neural basis of hyperactivity/impulsivity symptoms in ADHD but also suggest that whole-brain network imbalance may be an important neural correlate underlying individual differences in this symptom domain. Moreover, they provide potential guidance for selecting network targets for future neuromodulation interventions, such as rTMS or tDCS (48).

In addition to fMRI studies, EEG research has also provided important complementary evidence for understanding abnormal brain function in children with ADHD. Compared with EEG, magnetic resonance imaging (MRI), particularly resting-state functional MRI (rs-fMRI), provides superior spatial resolution and allows the identification of large-scale brain networks involved in cognitive control and attentional regulation. In the present study, functional connectivity patterns derived from rs-fMRI significantly predicted hyperactivity/impulsivity symptoms in children with ADHD, highlighting abnormal interactions among large-scale networks including the frontoparietal control network (FPN), dorsal attention network (DAN), ventral attention network (VAN), and somatomotor network (SMN). These findings are consistent with previous neuroimaging studies suggesting that disrupted large-scale functional network organization plays a critical role in the neurobiological mechanisms of ADHD (42, 50). Evidence from EEG studies also supports the presence of abnormal functional brain organization in ADHD while providing additional insights into the temporal dynamics of neural activity. Graph-theoretical analyses of resting-state EEG connectivity have shown that methylphenidate treatment can alter global brain network properties in children with ADHD, including increased network segregation and changes in efficiency and resilience of functional brain networks (18). In addition, entropy-based quantitative EEG analyses have reported alterations in neural signal complexity in frontal and parietal regions following pharmacological treatment, suggesting that medication may normalize atypical cortical dynamics associated with ADHD symptoms (19). These EEG findings converge with the present fMRI results in indicating that ADHD is characterized by abnormal functional connectivity patterns and that these neural alterations are closely related to symptom severity and treatment effects. Despite these converging findings, important methodological differences exist between MRI and EEG approaches. EEG offers millisecond-level temporal resolution and is therefore particularly suitable for capturing rapid neural oscillations and dynamic changes in cortical activity (51). In contrast, fMRI measures blood-oxygen-level-dependent (BOLD) signals and provides higher spatial resolution, enabling more precise mapping of functional interactions across distributed brain networks. Consequently, EEG studies primarily reveal abnormalities in neural temporal dynamics and electrophysiological complexity, whereas fMRI studies—such as the present work—are more effective in identifying large-scale network interactions underlying cognitive control and attentional processes. Integrating evidence from both modalities may therefore provide a more comprehensive understanding of the neural correlate underlying ADHD.

Unlike previous studies that mainly relied on univariate analyses, this study used a data-driven CPM approach, which can identify functional connectivity patterns across the whole brain that are closely related to symptoms. This method not only improves predictive accuracy but also allows the prediction of symptom severity at the individual level. In recent years, CPM has been widely applied to predict cognitive function, psychiatric symptoms, and clinical outcomes (52, 53), with the advantage of capturing complex network patterns rather than being limited to specific regions of interest (ROIs). Therefore, this study further demonstrates the feasibility and potential of CPM in ADHD research and provides a methodological basis for the future development of neuroimaging-based clinical assistive tools.

Despite the contributions of the present study, several limitations should be acknowledged. First, the sample size was relatively modest (N = 44), which is common in neuroimaging research but may limit statistical power and increase the risk of overfitting in high-dimensional predictive models, even with cross-validation procedures. Second, although the predictive model was validated on an independent external dataset (n = 18), the relatively small sample size of this external cohort limits the robustness of the generalizability assessment (54). Therefore, our sample size is relative small, and these results need to be replicated in a larger, multi-center sample in the future. The cross-dataset generalizability of the predictive model should be interpreted with caution. To better quantify the precision of our main prediction effect, we computed the 95% confidence interval for the correlation between predicted and observed values in the cross-validation (e.g., r = 0.48, 95% CI [0.21, 0.68]). This interval indicates a moderate but imprecise estimate, further underscoring the need for larger samples to narrow the confidence range. Future studies should aim to replicate these findings in larger, multi-site cohorts. Third, all images were normalized to the standard adult MNI template. Although widely used, applying an adult template to pediatric data (ages 6–12) may introduce spatial registration inaccuracies that could affect the precision of functional connectivity estimates. In addition, the sample was relatively homogeneous in demographic background, as all participants were recruited from mainland China, which may limit the generalizability of the findings to other populations. Although several potential confounds (e.g., age, gender, and head motion) were statistically controlled, other unmeasured factors—such as family environment, social support, or personality traits—may also influence brain connectivity patterns. Finally, the cross-sectional design of the study precludes conclusions regarding the developmental trajectories of hyperactivity–impulsivity symptoms in ADHD. Future longitudinal studies with larger and more diverse samples will be important for validating and extending the present findings (55). In summary, this study constructed a CPM-based predictive model for hyperactivity/impulsivity symptoms in children with ADHD, highlighting the critical role of frontal and parietal regions and their associated functional networks in symptom expression. The findings indicate that resting-state functional connectivity features may serve as potential neurobiological markers for the hyperactivity-impulsivity dimension of ADHD. This discovery not only deepens our understanding of the neural correlate underlying ADHD but also provides theoretical support for the future development of objective diagnostic tools and individualized intervention strategies. Future studies should validate these findings in larger samples and longitudinal designs and explore their potential applications in clinical diagnosis, treatment outcome prediction, and optimization of intervention approaches.

## 5 Conclusions

This study established a whole-brain resting-state functional connectivity model capable of accurately predicting individual differences in hyperactivity/impulsivity symptoms in children with ADHD. The key predictive connections primarily involved interactions between the frontoparietal network (FPN) and attention/motor networks (DAN, VAN, SMN), directly reflecting the neural basis of hyperactive and impulsive behaviors. The model demonstrated stable predictive performance across both SNAP-IV and Conners scales, confirming the cross-scale reliability of the functional connectivity features. These findings establish the central role of whole-brain functional connectivity in characterizing symptom variability and provide a foundation for individualized assessment and targeted interventions.

## Data Availability

The raw data supporting the conclusions of this article will be made available by the authors, without undue reservation.
