# Supplementary material for: A connectome-based neural correlate of pediatric ADHD hyperactivity–impulsivity symptoms
Source: Front Psychiatry. 2026 Jun 17;17:1846942. doi: 10.3389/fpsyt.2026.1846942 (PMC13318954; doi:10.3389/fpsyt.2026.1846942)
Supplement: Supplementary file 1 [file Supplementaryfile1.docx]

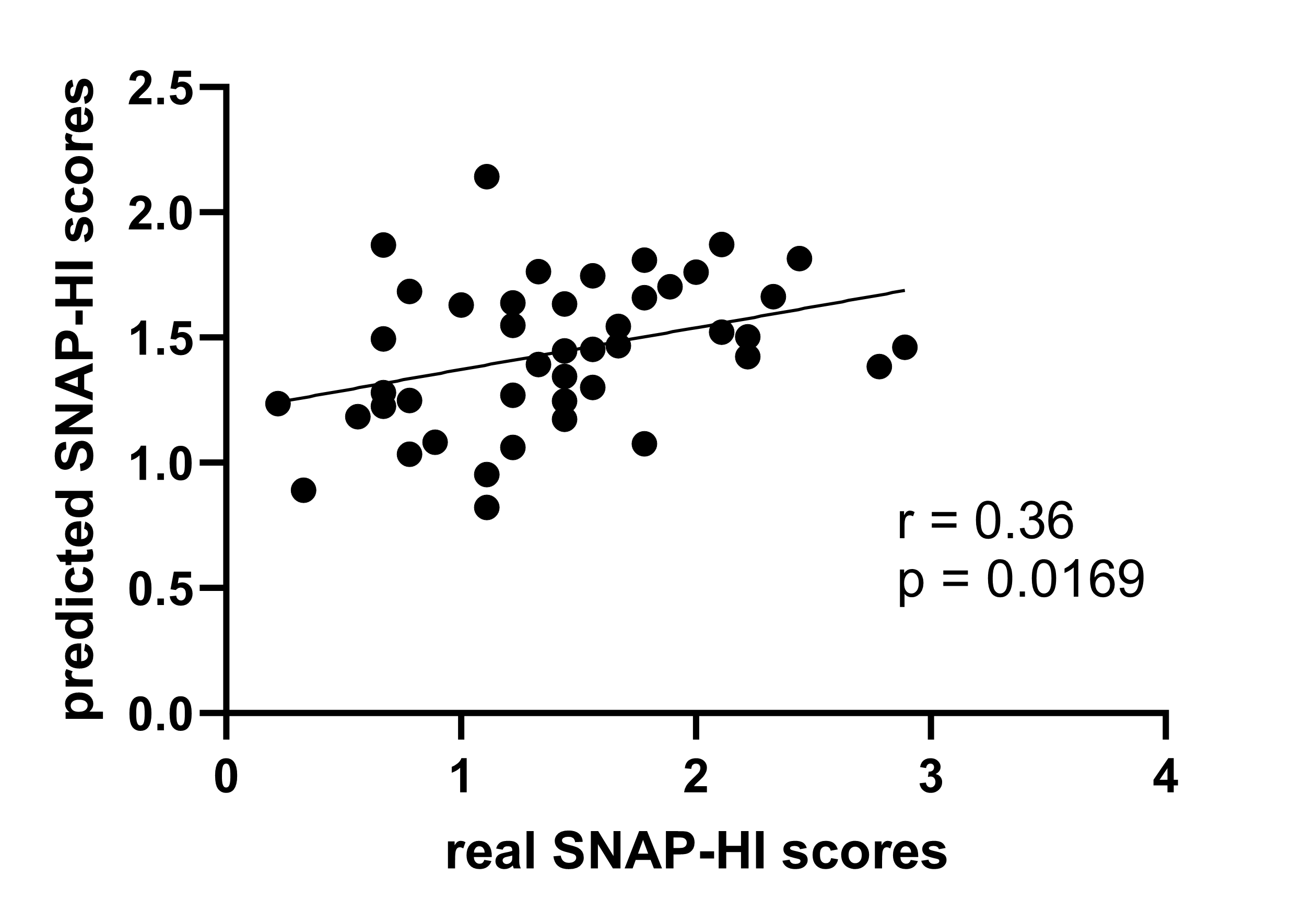


**Supplementary Figure 1.** Sensitivity analysis with additional control for socioeconomic status (SES) and intelligence scale (IQ).Partial correlation scatter plot between the observed SNAP-IV hyperactivity/impulsivity scores and the predicted scores after adjusting for age, sex, head motion, SES, and IQ. The displayed residuals are derived from regression models that regressed each variable against all covariates.
